# Negative Differential Conductance Induced by Majorana Bound States Side-Coupled to T-Shaped Double Quantum Dots

**DOI:** 10.3390/nano15171359

**Published:** 2025-09-03

**Authors:** Yu-Mei Gao, Yi-Fei Huang, Feng Chi, Zi-Chuan Yi, Li-Ming Liu

**Affiliations:** 1 School of Electronic and Information Engineering, UEST of China, Zhongshan Institute, Zhongshan 528400, China; yumeigao@zsc.edu.cn (Y.-M.G.); yizichuan@zsc.edu.cn (Z.-C.Y.); liulmxps@zsc.edu.cn (L.-M.L.); 2 School of General Education, Quanzhou Ocean Institute, Quanzhou 362700, China

**Keywords:** electronic transport, negative differential conductance, double quantum dots, Majorana bound states, zero-bias anomaly

## Abstract

Electronic transport through T-shaped double quantum dots (TDQDs) connected to normal metallic leads is studied theoretically by using a nonequilibrium Green’s function method. It is assumed that the Coulomb interaction exists only in the central QD (QD-1) sandwiched between the leads, and it is absent in the other reference QD (QD-2) side-coupled to QD-1. We also consider the impacts of Majorana bound states (MBSs), which are prepared at the opposite ends of a topological superconductor nanowire (hereafter called a Majorana nanowire) connected to QD-2, on the electrical current and differential conductance. Our results show that by the combined effects of the Coulomb interaction in QD-1 and the MBSs, a negative differential conductance (NDC) effect emerges near the zero-bias point, where MBSs play significant roles. Now, the electrical current decreases despite the increasing bias voltage. The NDC is prone to occur under conditions of low temperature, and both of the two QDs’ energy levels are resonant to the leads’ zero Fermi energy. Its magnitude, which is characterized by a peak-to-valley ratio, can be enhanced up to 3 by increasing the interdot coupling strength, and it depends on the dot-MBS hybridization strength nonlinearly. This prominent NDC combined with the previously found zero-bias anomaly (ZBA) of the differential conductance is useful in designing novel quantum electric devices, and it may also serve as an effective detection means for the existence of MBSs, which is still a challenge in solid-state physics.

## 1. Introduction

Majorana bound states (MBSs) are zero-energy quasiparticle states that were early predicted to appear in topological superconductors at boundaries or in vortices [1,2,3]. These localized excitations are of their own antiparticles, which means that they are neither bosons nor fermions but rather topologically protected objects called “non-Abelian anyons”. They are then immune to all local perturbations and are protected by the topological gap itself. This makes them a potential candidate in constructing qubits for a fault-tolerant topological quantum computer [4,5]. The MBSs were early observed experimentally in a semiconductor–superconductor nanowire with strong spin–orbit interaction under a magnetic field manifested as a zero bias conductance peak (ZBCP) [6,7] or fractional Josephson effect [8]. There are also some other solid-state platforms for creating MBSs such as coupling a conventional superconductor to a topological insulator [9,10] or conventional semiconductor [11], doped topological insulators [12], iron-based superconductors [13], quantum Hall analogs of spinless superconductors [14], superconducting topological crystalline insulators [15], and superconducting shells surrounding spin–orbit-coupled semiconducting nanowire cores [16]. A recent work proposed to generate MBSs in a superconductor/topological-insulator/superconductor hybrid system under a very weak magnetic field [17], which is fundamentally different from previous work relying on a strong magnetic field.

Currently, detecting MBSs remains challenging, since the localized non-topological states can mimic their signatures. Much theoretical and experimental work has been carried out to probe MBSs through various phenomena including the 4π periodic Josephson current phase in junctions between topological superconductors [18], enhancement or sign reversion of the thermopower in thermoelectricity [19,20,21,22,23], interaction between MBSs and microwaves [24,25,26,27], and ZBCP at the edges of the Majorana nanowires [6,7]. Among the above detection schemes for MBSs, ZBCP in electrical transport measurements is the most popular one. But ZBCP can also be induced by other mechanisms, and it was classified by Pan and Das Sarma as [28] good (by actual MBSs), bad (by Andreev bound states [29] which maybe called quasi-MBSs, produced by accidental quantum dots (QDs) in the nanowire), and ugly (by random disorder) ones. Therefore, researchers have been continuously putting forward other powerful approaches to verify the existence of MBSs, of which hybrid setups with MBSs side-coupled to QDs are attractive. QDs are zero-dimensional mesoscopic devices with adjustable energy levels, electron–electron Coulomb interactions, particle numbers and coupling strength to the external environment [30]. In a system composed of MBSs side-coupled to a QD, Liu predicted theoretically a half-maximum conductance, which originates from the half-fermionic nature of the MBSs and emerges when the QD’s energy level is aligned to the electrochemical potentials of the leads connected to the QD [31]. This is reliable evidence of the MBSs and has subsequently been observed in an experiment with a QD coupled to an InAs-Al nanowire [32].

**Figure 1:**
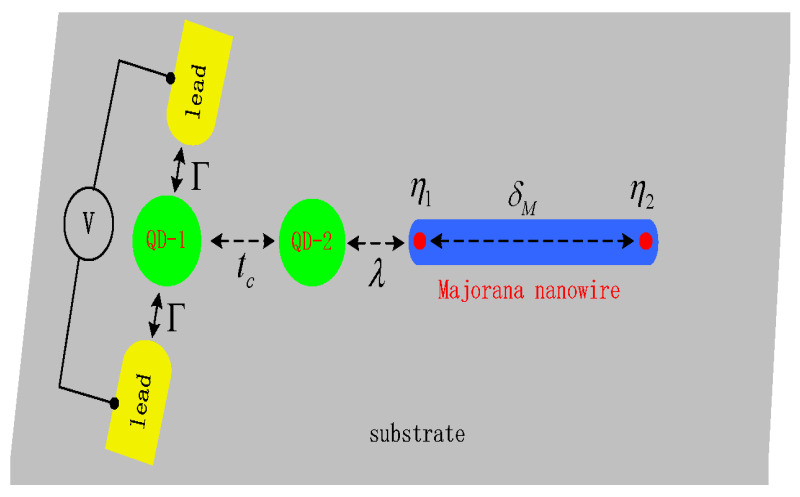
Schematic setup of T-shaped double quantum dots of which dot-1 is connected to the left and right metallic leads with equal strengthes Γ, and it tunnel couples to dot-2 with strength $t_{c}$. QD-2 is also side-coupled to a Majorana nanowire hosting MBSs (red circles at the ends of the blue nanowire), which are denoted individually by $\eta_{1}$ and $\eta_{2}$. The mode $\eta_{1}$ couples to QD-2 with strength of λ and to mode $\eta_{2}$ with strength of $\delta_{M}$.

Probing MBSs through differential tunneling conductance spectroscopy in hybridized QDs-MBSs system has been under intensive investigation in recent years [33,34,35,36]. Some works were also devoted to identifying the MBSs by combining electrical transport measurements with quantum interference effects in multiple-QD or multiple-path systems [37,38,39,40,41]. In such systems, both the peak’s height and positions of the differential conductance are varied by the coaction of MBSs and interference effects, and then they can be used for detecting the existence of MBSs or for preparing other functional electrical devices. In the present paper, we study electronic transport through T-shaped double QDs (TDQDs) in the presence of MBSs. As is shown in the schematic plot in Figure 1, the system is composed of QD-1 sandwiched between two normal metallic leads and QD-2, which is further side-coupled to one mode of the MBSs prepared at the ends of a Majorana nanowire. In particular, we focus on the phenomenon of negative differential conductance (NDC) induced by the MBSs and intradot Coulomb interaction in QD-1. The NDC refers to a decrease in current with increasing bias voltage, and it is unique in mesoscopic systems [42,43,44,45,46,47,48]. It has attracted much interest in view of its wide applications in designing various electronic devices, including logic circuits, fast switching, low-power memory, and various energy-saving devices [42]. The NDC has been observed in many devices such as monolayers [42,47], molecules [45,46], and systems based on semiconductor QDs [43,44,48]. The physical origins of the NDC effect may be attributed to features of the electrodes or the interface, bias-dependent tunnel-barriers, spin or phonon blockade, conformational switching [42], level spacing in the QDs or the hopping rate between the QDs [43], quantum interferences [44], etc. Currently, applications of NDC are hindered by two factors. One is the relatively small magnitude with a peak-to-valley ratio less than 2, and the other is the exhibition in a relatively high bias region that will consume more energy. Here, we find that the NDC generally occurs near a zero-bias point, and the peak-to-valley ratio can reach as large as about 3 when the central QD-1 is coupled weakly to the leads but strongly to QD-2, and thus it will be helpful to overcome the above two hinderments.

It is worth noting that the present system consisting of TDQDs has been extensively studied in previous work due to the particular transport behaviors in it. For example, if QD-1 is coupled to normal metallic leads, its density of states (DOS) has a broad resonance and develops a sharp dip structure due to the interference with the states from QD-2. In this case, the transport properties are determined by the Kondo effects in the presence of a Coulomb interaction in QD-1 [49], QD-2 [50] or both of the two dots [51,52] and the distinct Fano interference [53,54,55]. When QD-1 is coupled to one [56,57,58] or two superconductor leads [59,60], the interplay between the Kondo, Fano and Andreev effects exerts drastic influences on the transport processes. Recently, extensive investigations were also devoted to tunneling in TDQDs coupled to Majorana nanowires [37,39,61,62,63,64]. Now, the Majorana-mediated Kondo and Fano effects may serve as a reliable fingerprint for the presence of MBSs. In these studies, usually a linear response regime (infinitesimal bias voltage or temperature gradient) is considered, and the more realistic nonequilibrium cases [37,65] with finite applied bias voltage are neglected. Compared to those previous works, here we study the nonequilibrium differential conductance in TDQDs with MBSs prepared in the Majorana nanowire side-coupled to QD-2. The NDC induced by the coaction of MBSs and Coulomb interaction in QD-1 will be unique proof of the existence of MBSs.

## 2. Model and Method

The Hamiltonian of the present studied system can be written in the following form [31,37,62,63,64]

(1) $$\begin{array}{ccc} H & & =\sum_{k\alpha \sigma }\varepsilon_{k\alpha \sigma }c_{k\alpha \sigma }^{†}c_{k\alpha \sigma }+\sum_{i=1,2;\sigma }\left(\varepsilon_{i}+\xi eV\right)d_{i\sigma }^{†}d_{i\sigma }+Ud_{1↑}^{†}d_{1↑}d_{1↓}^{†}d_{1↓}+t_{c}\sum_{\sigma }\left(d_{1\sigma }^{†}d_{2\sigma }+H.c.\right)\\ & & +\sum_{k\alpha \sigma }\left(V_{k\alpha }c_{k\alpha \sigma }^{†}d_{1\sigma }+H.c\right)+H_{MBSs}, \end{array}$$

where $c_{k\alpha \sigma }^{†}$$\left(c_{k\alpha \sigma }\right)$ creates (annihilates) an electron with momentum *k*, energy $\varepsilon_{k\alpha \sigma }$, and spin direction σ=↑,↓ in the left and right leads (α=L,R). The operator $d_{i\sigma }^{†}$$\left(d_{i\sigma }\right)$ is the creation (annihilation) operator of an electron in dot-*i* with spin σ, and gate voltage tunable energy level $\varepsilon_{i}$, which may depend weakly on the bias voltage *V* [66]. In numerical calculations, we set ξ=0.05 for QD-1 and ξ=0 for QD-2 as only QD-1 is coupled to the leads directly. It is also assumed that there is an intradot Coulomb interaction *U* only in QD-1, which is accounted by the third term in Equation (1). The fourth term in Equation (1) describes an interaction between the two QDs with a strength of $t_{c}$. The coupling strength between QD-1 and lead − α is described by $V_{k\alpha }$. The last term $H_{MBSs}$ in Equation (1) is for the MBSs prepared at opposite ends of the Majorana wire [31,37,62,63,64]. Here, we consider the case that only one mode of the MBSs couples to the spin-up electrons in QD-2 due to the helical nature of the MBSs [31,39,62,63,64]. In practical devices, both spins of QD-2 may couple to the MBSs with different amplitudes due to the spin-mixing effects, disorder, or imperfect spin filtering [32,35,67]. These spin-dependent hybridization amplitudes depend on the distance between the Majorana nanowire and QD, and they can serve as a suitable tool to probe the topology of the Majorana wave function. If the system is under a strong enough magnetic field, however, there is only one spin-resolved energy level on the QD, and the present assumption is reasonable. The hybridization strength between QD-2 and MBS is denoted by λ, and the explicit expression of $H_{MBSs}$ is [31,37,62,63,64]

(2) $$\begin{array}{c} H_{MBSs}=i\delta_{M}\eta_{1}\eta_{2}+\lambda \left(d_{2↑}-d_{2↑}^{†}\right)\eta_{1}, \end{array}$$

in which $\delta_{M}$ is the interaction strength between the MBSs whose operators satisfy $\eta_{i}=\eta_{i}^{†}\left(i=1,2\right)$ and $\left\{\eta_{i},\eta_{j}\right\}=2\delta_{i,j}$. As usual, we rewrite $\eta_{j}$ in terms of the regular fermionic operators *f* as $\eta_{1}=\left(f^{†}+f\right)/\sqrt{2}$ and $\eta_{2}=i\left(f^{†}-f\right)/\sqrt{2}$, and then $H_{MBSs}$ is given by [31]

(3) $$\begin{array}{c} {\tilde{H}}_{MBSs}=\delta_{M}\left(f^{†}f-\frac{1}{2}\right)+\frac{\lambda }{\sqrt{2}}\left(d_{2↑}-d_{2↑}^{†}\right)\left(f^{†}+f\right). \end{array}$$

The spin-dependent current $J_{\sigma }$ tunneling between QD-1 and the leads is calculated in terms of the nonequilibrium Green’s function technique [31,37,68]

(4) Jσ=eh∫dε2πΓLΓRΓL+ΓR[−2ImG1σr(ε)][fL(ε)−fR(ε)],

where the line-width function $\Gamma \alpha =2\pi |V_{k\alpha }{|}^{2}\rho_{\alpha }$ where $\rho_{\alpha }$ is the DOS of electrons in lead − α. Under the wide-band approximation, $\rho_{\alpha }$ is independent of electron energy. The quantity $f_{\alpha }\left(\varepsilon \right)=1/\left\{1+\exp\left[\left(\varepsilon -\mu_{\alpha }\right)/k_{B}T\right]\right\}$ is the Fermi–Dirac distribution function in lead − α with chemical potential $\mu_{\alpha }$, temperature *T* and Boltzmann constant $k_{B}$, respectively. The term ImG1σr(ε) in the above equation is the imaginary part of the retarded Green’s function of QD-1, which is obtained by adopting the equation of motion method. Its explicit expression is give by [20,21,69]

(5) $$\begin{array}{c} G_{1\sigma }^{r}\left(\varepsilon \right)=\frac{\varepsilon_{1U}+U<n_{1\bar{\sigma }}>}{\varepsilon_{1U}\left[\varepsilon_{1-}-\Pi_{\sigma }\left(\varepsilon \right)\right]}, \end{array}$$

in which $\varepsilon_{1U}=\varepsilon_{1-}-U-t_{c}^{2}/\left(\varepsilon -\varepsilon_{2}+i0^{+}\right)$, and

(6) $$\begin{array}{c} \Pi_{\sigma }\left(\varepsilon \right)=\frac{t_{c}^{2}\left(\varepsilon_{2+}\varepsilon_{1+}-t_{c}^{2}\right)}{\varepsilon_{2-}\left(\varepsilon_{2+}\varepsilon_{1+}-t_{c}^{2}\right)-\varepsilon_{1+}K^{2}}, \end{array}$$

where $\varepsilon_{1\pm }=\varepsilon \pm \varepsilon_{1}+i\left(\Gamma_{L}+\Gamma_{R}\right)/2$, $\varepsilon_{2\pm }=\varepsilon \pm \varepsilon_{2}$ and $K=2\lambda^{2}\varepsilon /\left(\varepsilon^{2}-\delta_{M}^{2}+i0^{+}\right)$. Note that we have used the Hubbard-I truncation scheme for the higher-order Green’s functions, i.e., neglecting both the simultaneous tunneling of opposite spin direction electrons ($<d_{\sigma }d_{\bar{\sigma }}>=0$) and spin flip processes ($<d_{\sigma }^{†}d_{\bar{\sigma }}>=0$) on QD-1. Detailed calculation processes can be found in Ref. [69], and here we do not show them for the sake of conciseness. The electron occupation number $<n_{1\sigma }>$ in Equation (5) needs to be calculated self-consistently from the following equation [20,21,69],

(7) <n1σ>=∫dεΓLfL(ε)+ΓRfR(ε)ΓL+ΓR[−ImG1σr(ε)/π].

## 3. Numerical Results

**Figure 2:**
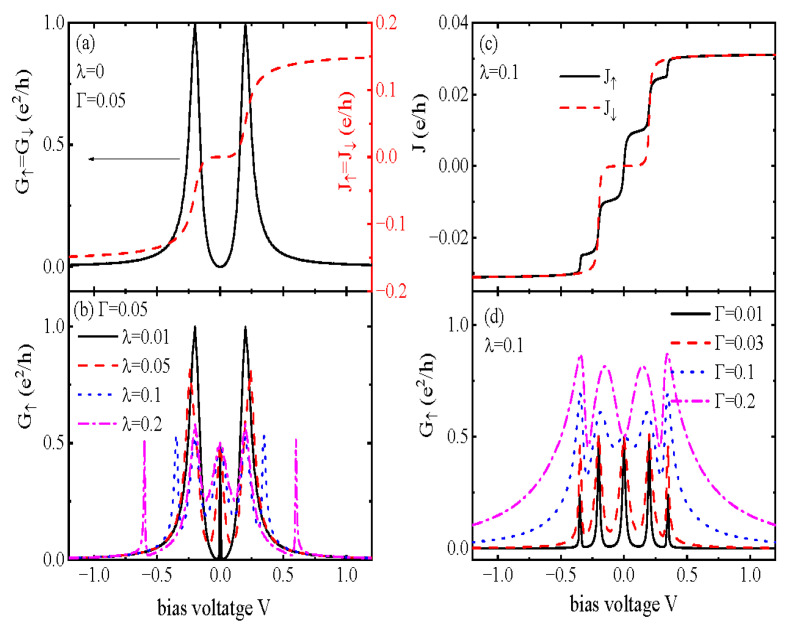
Spin-dependent current $J_{\sigma }$ in (**a**,**c**), and the corresponding differential conductance in (**b**,**d**) varying with the bias voltage in the case of intradot Coulomb interaction U=0. Now, the spin-down current and differential conductance are not affected by the MBSs, as only spin-up electrons in QD-2 couple to one mode of them. The energy levels of the two dots are all set to be zero ($\varepsilon_{1}=\varepsilon_{2}=0$). Other parameters are interdot coupling strength $t_{c}=0.1$, MBS–MBS hybridization amplitude $\delta_{M}=0$, and the system temperature T=0.

In numerical calculations, we set the intradot Coulomb interaction U=1 (except in Figure 2 where U=0) as the energy unit, and we consider that QD-1 couples to the two leads with equal strengths $\Gamma_{L}=\Gamma_{R}=\Gamma$. Figure 2 presents the electrical current $J_{\sigma }$ and the corresponding differential conductance $G_{\sigma }=dJ_{\sigma }/dV$ varying with the applied bias voltage in the absence of the Coulomb interaction (U=0). Under these conditions, our results are essentially consistent with those of Gong et al. [37]. For λ=0, the electrical current $J_{↑}=J_{↓}$ increases whenever the chemical potentials of the leads $\mu_{L}$ and $\mu_{R}$ are aligned to the molecular states $\varepsilon =\pm t_{c}$ for the dots’ levels $\varepsilon_{1}=\varepsilon_{2}=0$, which is indicated by the red dashed line in Figure 2a. Here, we assume that the bias voltage is applied across the system symmetrically, i.e., $\mu_{L}=-\mu_{R}=eV/2$; then, the differential conductance develops peaks at $eV=\pm 2t_{c}$ as shown by the black solid line in Figure 2a [37,63,64]. We next show the spin-up conductance $G_{↑}$ in Figure 2b when QD-2 interacts with the MBS prepared at the end of the Majorana wire (λ≠0). The spin-down conductance is the same as that in Figure 2a in the case of U=0. Figure 2b shows that the peaks in $G_{↑}$ originally positioned at $\pm 2t_{c}$ are suppressed and then split for a large enough λ, as indicated by the blue dotted and purple dash–dotted lines. More interestingly, a new peak emerges at the zero-bias point (V=0) which is the previously found ZBA induced by MBSs [31,37]. The peak’s height of $G_{↑}{|}_{V=0}$ is exactly equal to $e^{2}/\left(2h\right)$, which shows the half-fermion nature of the MBSs. This result agrees with those found in either single QD [31,68] or TDQDs [37,62,63,64] structures in the presence of MBSs. Since the height of the ZBA is completely unaffected by the value of the MBS-QD hybridization strength, it can be viewed as evidence of the existence of MBSs. If the Majorana nanowire is replaced by a conventional superconducting nanowire, Andreev reflection processes will also induce peaks in the differential conductance around the zero-bias point [70]. The peak height in this case, however, depends on the hybridization strength between the QD and the superconductor, which is quite different from the present result. Figure 2c,d show the current $J_{\sigma }$ and the associated differential conductance $G_{\sigma }$ as functions of the bias voltage for fixed λ=0.1 and different values of Γ. As usual, the current in Figure 2c increases whenever $\mu_{L/R}$ is aligned to the molecular states $\pm t_{c}$, resulting in peaks in $G_{\sigma }$. The peaks become wider and higher with increasing Γ due to the stronger interaction between QD-1 and the leads.

**Figure 3:**
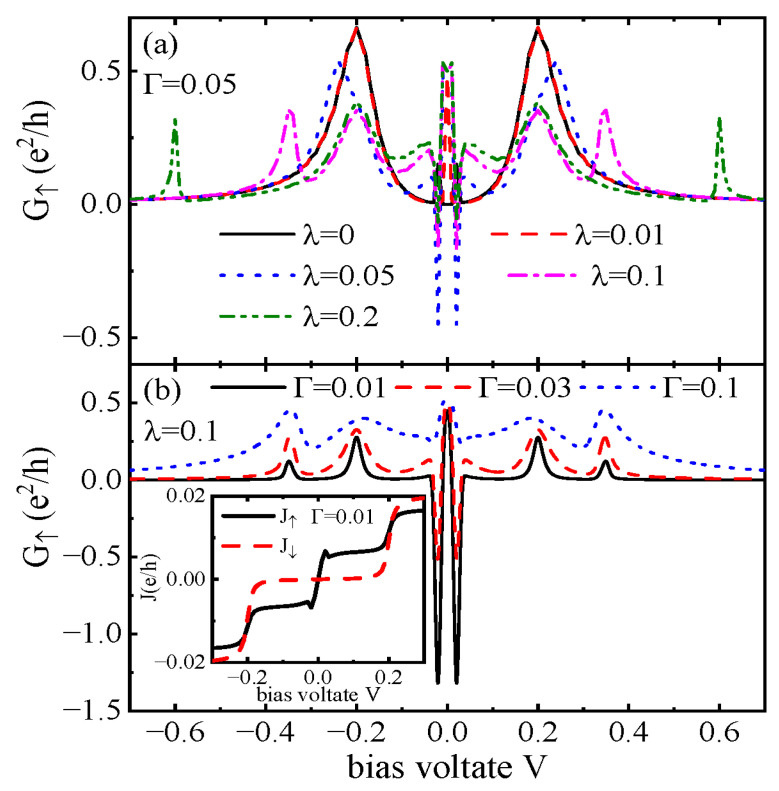
Spin-up differential conductance $G_{↑}$ as a function of the bias voltage *V* for U=1 and different λ in (**a**) and different Γ in (**b**). The inset in (**b**) shows the spin-resolved current varying with the bias voltage with λ=0.1 and Γ=0.01. Other parameters are as shown in Figure 2.

In Figure 3, we present the spin-up differential conductance $G_{↑}$ for fixed intradot Coulomb interaction U=1. Under this condition, although the MBSs also affect the transport processes of the spin-down electrons, the impacts are relatively weak and then we do not show $G_{↓}$ here. If QD-2 is free from coupling to the MBS (λ=0), the Coulomb blockade effect will induce a pair of peaks in $G_{↑}$ around eV=±2U, which are less affected by the MBSs. The peaks in $G_{↑}$ at $\pm 2t_{c}$ previously shown in Figure 2 still survive, as indicated in Figure 3a. Different from the case of U=0, another pair of peaks emerge around eV≈±Γ due to the shift of the molecular states in the presence of a finite Coulomb interaction in QD-1. Turning on coupling between QD-2 and the MBS (λ≠0), the peaks around $\pm 2t_{c}$ are lowered and split, as seen in those in Figure 2b. The ZBA with a fixed height of $e^{2}/2h$ emerges again due to the hybridization between QD-2 and the MBS. As a result, the original peaks around eV≈±Γ are suppressed, and they can even become negative by increased λ, which is an interesting NDC phenomenon. The magnitude of the NDC depends nonlinearly on the value of λ. For λ∼Γ, the absolute value of NDC has a maximum of $|G_{↑}|\approx e^{2}/2h$, and then it decreases with increasing λ. Figure 3b shows that $G_{↑}$ is sensitive to the value of Γ. At weak coupling between the the leads and QD-1, the NDC is obviously enhanced with $G_{↑}\left(eV\approx \pm \Gamma \right)$ and can reach about $-1.25e^{2}/h$. This can be seen from the inset in Figure 3b in which $J_{↑}$ indicated by the solid black line decreases with increasing bias voltage around eV≈±Γ. The spin-down current varies smoothly at eV≈±Γ. Comparing the behaviors of $J_{↑}$ and $J_{↓}$, the NDC can be attributed to the existence of MBSs. Increasing the value of Γ, the magnitude of NDC is weakened accompanied by the widening of other peaks. For large enough Γ=0.1, the NDC disappears as indicated by the blue dotted line in Figure 3b as the functions of the MBS are suppressed when electrons are strongly coupled to the leads.

**Figure 4:**
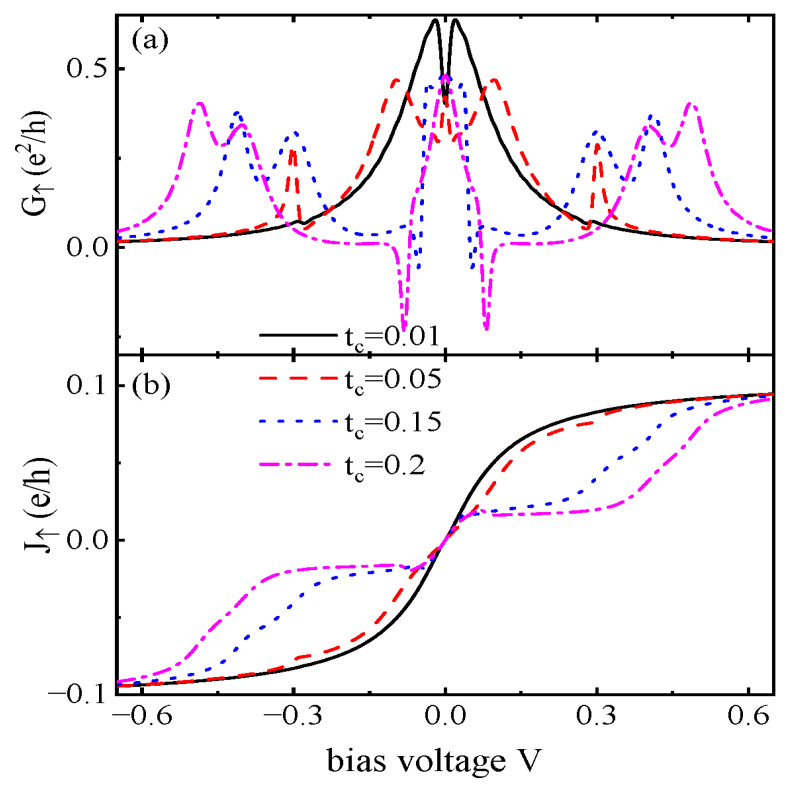
(**a**) Spin-up differential conductance $G_{↑}$ and (**b**) spin-up current $J_{↑}$ as functions of the bias voltage *V* for U=1, Γ=0.05, and different values of interdot coupling strength $t_{c}$. Other parameters are as shown in Figure 2. Note that the NDC is enhanced by increasing $t_{c}$.

Figure 4 presents the dependence of $G_{↑}$ and $J_{↑}$ on the interdot coupling $t_{c}$ with fixed λ=0.1. For weak interdot coupling $t_{c}=0.01$, the height of the ZBA induced by MBS is fixed at $e^{2}/2h$, and the other two peaks with positive values are located about at $eV=\pm 2t_{c}$. With an increasing value of $t_{c}$, the peaks are lowered and split, which is similar to the cases in Figure 2 and Figure 3, except for those of the ZBA. When the interdot coupling is strong enough, NDC emerges, as is indicated by the blue dotted and purple dash–dotted lines in Figure 4a. The magnitude of the NDC is enhanced by increasing $t_{c}$ as the impact of the MBS is strengthened. Meanwhile, it is found that the peaks at higher energy states $\varepsilon \approx \pm \sqrt{t_{c}^{2}+\lambda^{2}}$ in Figure 4a are further split and become higher. This is because the value of the original conductance peaks around ±Γ are changed from positive to negative, inducing the NDC phenomenon. Now, more electrons will be transported through the system from the energy states of $\pm \sqrt{t_{c}^{2}+\lambda^{2}}$ and result in increased $G_{↑}$. Figure 4b shows that the magnitude of $J_{↑}$ is weakened by increasing $t_{c}$ due to the quantum interference processes. For $t_{c}=0.15$ and 0.2, $J_{↑}$ decreases with increasing bias voltage around eV≈±Γ and results in the phenomenon of NDC. In contrast, the spin-down current $J_{↓}$ will increase obviously at the molecular states of $\pm \sqrt{t_{c}^{2}+\lambda^{2}}$, and this leads to peaks in the differential conductance. Apart from these two states, $J_{↓}$ will change smoothly, and no peak is induced in the differential conductance.

**Figure 5:**
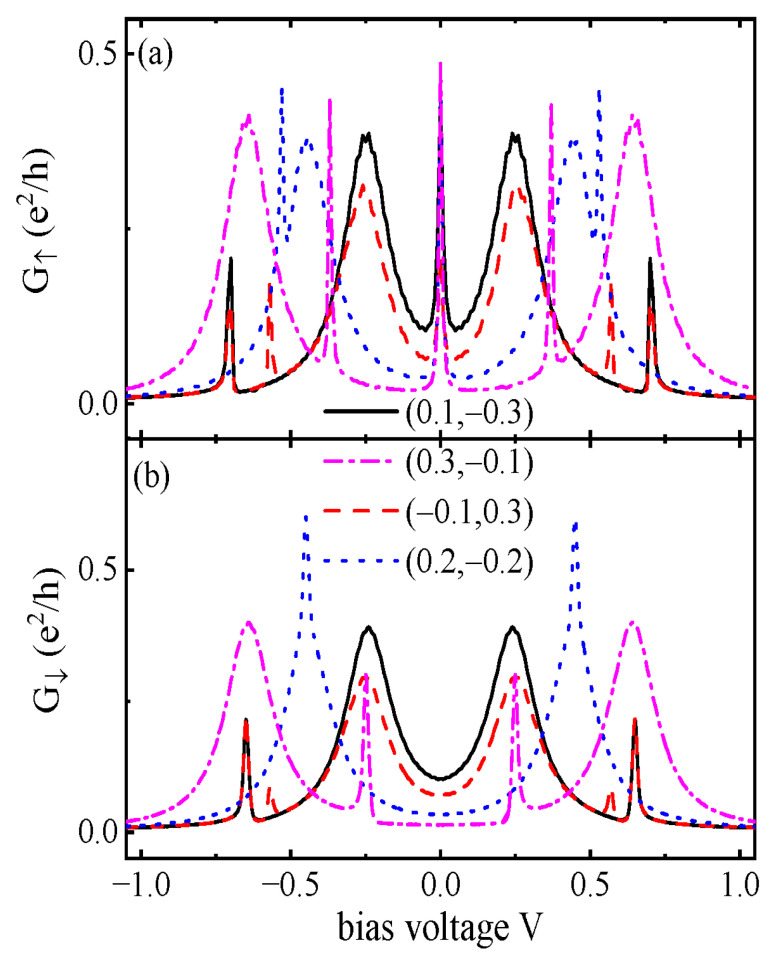
(**a**) $G_{↑}$ and (**b**) $G_{↓}$ as functions of the bias voltage *V* for different configurations of the dots’ levels $\left(\varepsilon_{1},\varepsilon_{2}\right)$ for U=1, Γ=0.05. Other parameters are as shown in Figure 5.

In Figure 5, we examine the impacts of QDs’ energy levels’ configuration $\left(\varepsilon_{1},\varepsilon_{2}\right)$ on the differential conductance. Figure 5a shows that the ZBA in $G_{↑}$ with a height of $e^{2}/2h$ is essentially unchanged by the variation in the dots’ energy levels, showing reliable evidence for the existence of MBSs. Apart from the zero-bias point, both the position and height of the conductance peaks are sensitive to the configuration of the QDs’ energy levels $\left(\varepsilon_{1},\varepsilon_{2}\right)$. Importantly, the NDC disappears when the QDs’ energy levels are shifted away from the Fermi level of the leads $\mu_{F}=0$ as the molecular states and quantum interference processes are changed. In experiments, the energy levels of the QDs can be adjusted by external gate voltages or the dot’s shape in a wide range [30,32]. Moreover, as the MBSs usually manifest them around the zero bias regime, then the zero dots’ energy levels may be even desirable. As for the spin-down differential conductance $G_{↓}$ in Figure 5b, it has a dip whose value can be suppressed to zero at eV=0 due to the Fano antiresonance. The peaks of $G_{↓}$ at other bias voltages are essentially the same as those of $G_{↑}$ as the zero-energy modes of MBSs mainly change transport processes around the Fermi energy levels.

**Figure 6:**
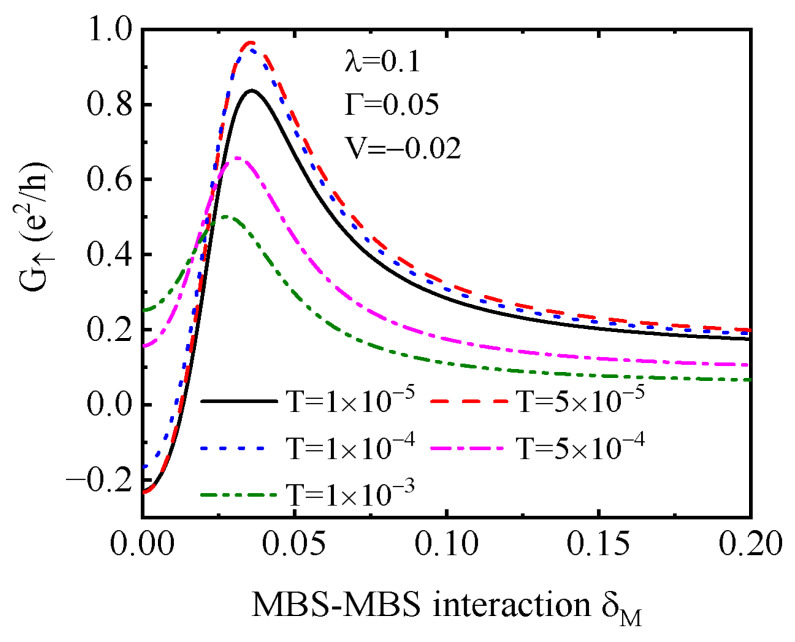
Dependence of $G_{↑}$ at V=−0.02 around which NDC emerges on the MBS–MBS coupling strength $\delta_{M}$ for different system temperature *T* and indicated parameters. Other quantities are U=1 and $t_{c}=0.1$.

The two modes of the MBSs $\eta_{1}$ and $\eta_{2}$ couple to each other with strength of $\delta_{M}∼e^{-L/\zeta }$, where *L* is the length of the Majorana nanowire and ζ is the superconducting coherence length [31,32,35]. In Figure 6, we present functions of $\delta_{M}$ on the NDC in $G_{↑}$ for different values of the system temperature *T*. It shows that if the nanowire is long enough so that $\delta_{M}\ll \lambda$, one can observe the NDC at low temperatures (see the black solid, red dashed and blue dotted lines). When the temperature is increased to $5\times 10^{-4}$ and $1\times 10^{-3}$, $G_{↑}$ becomes positive and the NDC disappears. The reason is that the peaks in the transmission coefficient are broadened, and the impacts of the MBS are eliminated by the thermal motion of the electrons at high temperatures. In experiments, the intradot Coulomb interaction *U* is well adjustable. For a typical value of U=10 meV [30], temperature $T∼10^{-4}$ in units of *U* corresponds to $k_{B}T∼10^{-3}$ meV and then $T∼10^{-2}$ K. Under this condition, the MBSs are realizable in topological superconductors $Sr_{2}RuO_{4}$ [3] or $Sn_{4}Au$ [71] with a very low critical temperature $T_{c}$ of several Kelvin. With increasing $\delta_{M}$, $G_{↑}$ first increases, reaching a maximum, and then it decreases to a constant value. The reason is that when the two modes of the MBSs overlap strongly with each other, they behave like the usual Dirac fermion [31]. In fact, even the height of the ZBA in the differential conductance $G_{↑}$ will also be changed in the presence of finite $\delta_{M}$, and the signature of the MBSs disappears [31].

## 4. Summary

In summary, we have studied quantum transport through TDQDs in which the side-coupled QD-2 is hybridized to a Majorana nanowire. It is found that due to the combined actions of the Coulomb interaction in QD-1 and the MBSs, the differential conductance of the spin-up electrons which couples to the MBSs is obviously changed. It can even be suppressed to a negative value under weakly applied bias voltage. This is the interesting NDC phenomenon unique in mesoscopic systems. The NDC is prone to occur under the conditions of strong interdot coupling and low temperatures, and it depends on the QD-MBS hybridization strength nonlinearly. The overlap between the MBSs will destroy the NDC, and it changes the MBSs into a usual Dirac fermion. The NDC as well as the ZBA in these TDQDs may serve as reliable evidence of the existence of MBSs, and they may also be useful in designing particular mesoscopic electronic devices.

## Data Availability

All data included in this study are available upon request by contact with the corresponding author.
